# Improving Mass Casualty Event Response: A Simulation-Based Program in a Rural Hospital

**DOI:** 10.7759/cureus.91025

**Published:** 2025-08-26

**Authors:** Hadas Katz-Dana, Rotem Shiri, Eran Netzer, Elad Dana, Jabeen Fayyaz, Ehud Rosenbloom

**Affiliations:** 1 Pediatric Emergency Medicine, Meir Medical Center, Kfar Saba, ISR; 2 Clalit Health Services, Simultech Center for Medical Simulation, Kfar Saba, ISR; 3 Anesthesiology, Intensive Care, and Pain Medicine, Meir Medical Center, Kfar Saba, ISR; 4 SickKids, University of Toronto, Toronto, CAN

**Keywords:** clinical simulation, disaster medicine, preparedness, rural, simulation, simulation-based education

## Abstract

Introduction: Mass casualty events (MCEs) pose significant challenges, especially for hospitals serving remote populations. The increasing threat of modern terrorism underscores the importance of robust emergency preparedness. Simulation-based education (SBE) is an effective training approach to identify and address improvement areas. In October 2023, an urgent need arose to prepare a rural hospital in southern Israel for potential MCEs.

Methods: A simulation-based training program was developed for the hospital, focusing on trauma procedures, individual patient care, and team-based MCE drills. The training was implemented in three phases, addressing communication, procedural challenges, and operational/logistical gaps. Each simulation concluded with a debriefing session, followed by an after-action report and an improvement plan.

Results: Eighty healthcare providers participated in the training. The simulations revealed significant gaps in trauma procedural skills, communication, and logistical protocols. As a result, the hospital's preparedness was significantly enhanced, with key improvements in coordination and operational readiness for potential disasters."

Conclusions: Targeted SBE disaster preparedness programs in rural hospitals can improve trauma skills and MCE management while identifying latent safety threats. Continued investment in SBE is recommended to strengthen emergency response capabilities in similar settings.

## Introduction

A mass-casualty event (MCE) is defined as an incident in which medical services resources are overwhelmed by the number and/or severity of casualties, thus shifting the focus of medical care from the individual patient to the population as a whole in order to maximize survival [1]. The phenomenon of modern terrorism, in which one or a group of terrorists target a large population, has steadily become more commonplace, peaking in 2014 with over 16,000 terrorist incidents worldwide causing over 40,000 fatalities [2]. MCEs cause significant disruption to healthcare facilities, challenging both staff and resources due to their magnitude, unpredictability, and medical and logistic complexity [3].

Emergency preparedness training is critical in order to provide adequate quality of care in case of MCEs [4-8]. Simulation-based education (SBE) is distinctly beneficial in preparing for MCEs and is therefore routinely used with documented success across health professions [9]. SBE is effective in identifying latent gaps in translating the MCE preparation plan into action and allows frontline staff to familiarize themselves with local protocols and understand their role [10-12].

Rural hospitals face several unique challenges in disaster preparedness; allocating staff for training and planning is notably difficult due to limited personnel. Additionally, these hospitals are often remote from any external training team or facility, resulting in higher costs for self-purchased equipment and supplies without volume discounts. Finally, serving smaller, often low-income communities, overall preparedness efforts are further hindered by limited expenditure on potential events and focusing on more urgent needs [13-15].

This article outlines the key components of a mass casualty preparedness program implemented at Yoseftal Hospital, a rural hospital in southern Israel, during the October 2023 war. With thousands of displaced civilians arriving in the city of Eilat, the local population suddenly doubled [16]. In response to this sudden growth and escalating threats, a call to prepare for a potential MCE was issued. The situation was further complicated by the fact that Yoseftal, the only hospital in the city and the surrounding 200-kilometer radius, is the smallest general hospital in Israel, with a capacity of just 65 beds, four operating rooms, and a medical staff of approximately 25 specialists, 15 residents, and 120 nurses.

In response to this urgent need, the Meir Medical Center Academy for Emergency Medicine (MAEM), specializing in emergency care and trauma management training, was enlisted by national health authorities to develop a concise and effective preparedness program. The objective was to enhance Yoseftal Hospital's disaster response capabilities in these unprecedented circumstances.

This article details the inception and execution of this program, with the aim of informing similar efforts in the future.

## Materials and methods

In October 2023, the national health department conducted a comprehensive mass casualty event exercise at Yoseftal Hospital (pre-intervention phase). Insights from this exercise, assessed by independent evaluators affiliated with the health department, served as a needs assessment for the MAEM team to formulate a simulation exercise program tailored for the hospital. Subsequently, a dedicated working group, comprising key stakeholders from the hospital, was established to collaboratively design the most fitting intervention for optimal preparedness.

For the intervention, operation-based exercises (OBEs) were chosen. OBEs are conducted in real time, under realistic conditions that aim to replicate the true environment and actual stressors typical of an emergency. The main advantages of OBEs include improved knowledge of emergency plans and providers [17], improved monitoring of planning and training adequacy, and improvements in teamwork and network effectiveness, while reducing the level of stress of participants [18]. On an organizational level, OBEs help to identify gaps and limitations in emergency response plans, protocols, procedures, and training [7].

The simulation training program was performed in three phases (Table 1). Phase 1 focused on trauma skills and management. A single-day workshop was conducted four times over four consecutive days with up to 20 participants in each session, ensuring effective hands-on training. Additionally, participants attended a crisis resource management (CRM) lecture, focusing on effective communication, leadership, and situational awareness.

**Table 1:** Overview of the simulation-based MCE preparedness program MCE: mass casualty event

Phase	Focus	Description	Duration	Participants per day
Pre-intervention	External evaluation by the Ministry of Health	Independent MCE drill for outcome evaluation	One day (Day 1 )	Ministry-led assessment
Phase 1	Trauma skills and individual patient simulation	Hands-on workshops	Four days (Day 2–5)	Up to 20 per day
Phase 2	Medium-scale MCE simulations	Six site-specific MCE scenarios debriefings and improvement planning	Four days (Day 6–9)	
Phase 3	Hospital-wide full-scale MCE simulation	Large-scale exercise, 45 simulated patients across all hospital divisions	One day (Day 10)	Full hospital team
Post intervention	External evaluation by the Ministry of Health	Independent MCE drill for outcome evaluation	One day (Day 11)	Ministry-led assessment

To enhance proficiency in trauma skills tasks, trainers across four distinct modules were employed. Each module began with an explanation and demonstration of the procedure, followed by 15 minutes of practice per participant. Table 2 offers a synopsis of the content covered in each module. This was followed by trauma simulations focusing on the management of a single trauma patient in groups comprised of six team members (two physicians and four nurses). Each group engaged in two scenarios featuring a blunt trauma patient and a penetrating trauma patient. Following each scenario, facilitators conducted debriefing sessions.

**Table 2:** Summary of trauma skills modules

Skill	Description	Key challenges
Hemorrhage control	Placing a tourniquet, applying direct wound pressure, and wound packing	Recognizing hemorrhagic shock, awareness of tourniquet conversion times
Airway management	Repositioning maneuvers, mask ventilation, intubation, use of intubation aids (bougie)	Proficiency in difficult airway management
Chest decompression	Needle decompression, chest drain insertion	Rapid recognition of indications for chest decompression, familiarization with updated guidelines
Surgical airway	Cricothyroidotomy	Rarely used, resulting in a lack of experience with the procedure and equipment

Phase 2 consisted of six medium-sized MCE simulations. In alignment with real-life MCE response protocols, patients were placed into three distinct zones based on injury severity (severe, medium-severity, and holding area for evacuation). Two MCE scenarios were conducted for each zone, each lasting 1.5 hours, followed by a 45-minute debriefing session. The main scenario was of a terrorist attack on a city hotel, resulting in a significant number of victims ranging from eight to 15 at each site. Standardized patients (SPs) or high-fidelity manikins were used to represent victims. To enhance authenticity, comprehensive briefings were systematically provided to all SPs, allowing them to appropriately present symptoms concordant with their injury. SPs also received a safety briefing before participating in the simulated scenarios. Each patient was accompanied by a designated facilitator who monitored and, if needed, guided the active involvement of the patient and ensured a seamless simulation experience.

The MCE simulations actively engaged multidisciplinary teams, including medical and nursing staff, hospital logistics personnel, blood bank staff, and members of the imaging department. The teams operated as organic units, mimicking real-world coordination and collaboration expected in an actual disaster. Hospital stakeholders, including senior management, participated as observers, contributing to the debriefing sessions and development of an improvement plan. At the end of each scenario, a structured debrief was executed based on the Promoting Excellence and Reflective Learning in Simulation (PEARLS) debriefing framework [19]. These sessions served as a source of qualitative feedback and consistently revealed enhanced confidence and perceived preparedness among staff members in managing MCE scenarios.

The insights gained from Phase 2 were compiled into an after-action report, which led to revisions in the hospital's MCE protocol. These findings also informed the planning of Phase 3.

Phase 3 incorporated a hospital-wide, large-scale MCE simulation. During this phase, all relevant hospital divisions, including the MCE patients’ sites, trauma bay staff, blood bank, operating room, diagnostic imaging, and medical evacuation teams, took part. This scenario started with a major terrorist attack resulting in the gradual arrival of 45 patients to the hospital. Simulated data for 45 patients were generated. Patients were classified into priority categories: 20 priority one (P1) patients (immediate life threat), priority two (P2) patients (urgent care required), and five priority three (P3) patients (expected to die from injuries). The patients arrived over a duration of two hours, mirroring real-time patient influx. Patient flow was subjected to the treating team's decisions and could include imaging, operating room interventions, evacuation, or placement in the mortuary. The simulation exercise adhered to predefined time-linked endpoints, after which a comprehensive debriefing session occurred, facilitated by a clinician specialized in team debriefing.

Of the 45 simulated patients, 37 were SPs, and seven were simulated using advanced high-fidelity manikins. In cases where invasive procedures were warranted for an SP (e.g., chest drain insertion or intubation), high-fidelity task trainers were employed to replicate the procedure. Skilled trauma physicians with expertise in mass casualty management, including prior experience with MCEs (civilian or during military service), emergency medicine physicians, and simulation experts served as facilitators. The hospital and national health system stakeholders assumed the role of observers during the scenario, actively participating in subsequent debriefing sessions.

A week after the completion of Phase 3, a second comprehensive full-scale drill was conducted by the health department as a post-intervention evaluation phase.

## Results

A total of 80 physicians and nursing staff of various disciplines went through the training in a total of 11 days. Participants were comprised of 20 staff physicians (four surgeons, three anesthesiologists, three pediatricians, two emergency medicine physicians, two gynecologists, one ENT physician, one ophthalmologist, and four internal medicine physicians), 15 residents, and 45 nurses. All simulation exercises were followed by debriefings based on the PEARLS debriefing framework and conducted by simulation educators. During the debriefings, participants across all professional roles reported a significant improvement in their confidence to manage mass casualty events, including technical skills, communication, and coordination. Each simulation exercise uncovered latent errors and system safety threats that were noted in the debriefs (Table 3), including communication issues, role allocation, equipment, and area coordination. These were outlined in an after-action report and integrated into an improvement plan.

**Table 3:** Latent errors and gaps with suggested action items for intervention

Area of focus	Issues identified	Intervention
Communication	Lack of closed-loop communication, unclear hierarchy between team leader and team member, conflicting priorities resulting in delay or non-response	Implementation of closed-loop communication protocols, clarification of roles and authority structures, prioritization training, and streamlined communication processes
Task-specific issues	Lack of a detailed massive transfusion protocol, unclear pathway to obtain imaging, and team members unfamiliar with tourniquet conversion guidelines	Development and implementation of a comprehensive blood transfusion protocol, introduction of a streamlined pathway for imaging requests, integration of tourniquet conversion protocol into team training
Equipment and supplies	Shortages, including beds, IV poles, and medical supplies	Improved inventory management and increased equipment availability
Transport	A single elevator leading to helicopter evacuation delays	An alternative route to the evacuation site

The hospital's improvement in managing an MCE is demonstrated by the change in the Ministry of Health’s reports before and after the intervention. The reports, summarized in Table 4, show notable advances in different areas between the initial and the follow-up intervention evaluations.

**Table 4:** Key points of pre- and post- intervention reports MCE: mass casualty event

Pre-intervention report	Post-intervention report
Lack of communication between the patient site’s team leaders and the MCE leader, absence of blood product order forms, the holding area was not activated, and teams lacked confidence in their familiarity with trauma protocols; in conclusion, multiple gaps were identified.	Effective closed-loop communication, blood products were requested and effectively supplied through appropriate forms, the holding area was activated and functioned well as a station prior to surgery, hospitalization, or transfer; and the teams demonstrated a proficient understanding of trauma protocols; in conclusion, major improvement was seen in the team’s management of MCE.

## Discussion

This report outlines a successful simulation-based MCE preparedness program planned and implemented in a rural hospital in Israel during a time of national crisis. Our primary objective in sharing this experience is to inform future efforts in preparing for MCEs. After conducting a collaborative needs assessment, the program was developed to identify and address both medical and logistical gaps in MCE management. 

Insights for the intervention were initially derived from a pre-intervention evaluation conducted by the Ministry of Health. While such assessments may sometimes be perceived as "tests" that could pose challenges, particularly in hierarchical systems, we emphasize the critical need for such evaluations, especially when rapid outcomes are necessary. While pre- and post-intervention surveys were not feasible due to the urgent nature of implementation during an ongoing national emergency, the structured debriefings conducted after each simulation provided real-time qualitative assessments of participant experiences and perceived growth. Additionally, a full-scale MCE simulation conducted by the Ministry of Health one week after the intervention served as an external evaluation. This drill indicated significant improvements compared to a similar exercise conducted before the program, offering a practical benchmark of the intervention’s effectiveness.

A major outcome that remained relevant through the process was communication within and between teams. Effective communication is essential in the context of MCE. To address this issue, we integrated both physical measures (direct lines of communication between sites and additional devices) and appropriate protocols. The most commonly used model in this request is the CRM, which emphasizes closed-loop communication, role clarity, and situational awareness, and was a key tool in improving communication [20]. Communication significantly improved from phase 1 to phase 3, despite increasing scenario complexity.

Our findings align with prior research demonstrating the effectiveness of simulation-based exercises for disaster preparedness. Klima et al. demonstrated how full-scale regional simulations improve communication and coordination, both areas that showed measurable improvement in our program [12]. Similarly, the Skryabina et al. study examining emergency preparedness exercises in the context of a mass casualty terrorist incident emphasized the critical role such exercises play in enhancing communication, clarifying roles, and strengthening system-wide readiness, further supporting the effectiveness of our approach [21]. In a study of rural hospitals, Gillett et al. showed that simulations could be feasibly deployed in resource-limited environments, as in our study [11]. Moreover, our staged approach aligns with the Disaster Day model described by Gable et al., which emphasized repeated exposure and increasing complexity as essential to preparedness [6]. Finally, the detection of latent threats, such as the lack of a massive transfusion protocol, mirrors the results of Wetzel et al., who used simulation to identify safety threats in high-risk environments [21]. These findings underscore that simulation not only prepares individuals but also strengthens institutional readiness and reveals system-wide deficiencies.

Our program also uncovered significant latent safety threats, defined as errors in design, organizing, or training that could lead to medical errors and have a significant impact on patient safety [22]. For example, during phase 1, it became evident that no massive blood transfusion protocol exists, causing significant delays in providing this potentially life-saving intervention. Through an elaborate after-action report, this was effectively identified and addressed, with relatively minimal resource investment and resulting in major benefit.

While the intervention was successful, several limitations must be acknowledged. First, the intervention was conducted at a single rural hospital, which limits the generalizability of the findings. Second, the absence of a control group prevents us from directly measuring the impact and effectiveness of the program. Third, the intervention did not capture long-term outcomes or the retention of skills and knowledge by participants, which would require follow-up assessments and simulations. Finally, due to the urgent nature of the situation, we were unable to include quantitative evaluations, relying instead on structured debriefings and external assessments for outcome evaluation.

Based on our experience, we offer several recommendations for similar SBE programs in rural hospitals. First, a thorough needs assessment should be conducted before any intervention, followed by the development of a detailed action plan. Simulation exercises should be multidisciplinary, scenario-based, and involve multiple departments to reinforce skills and knowledge. Regular simulations are essential to maintain preparedness. To ensure more robust evaluations, objective measures such as checklists, debriefing tools, and clinical outcomes should be integrated alongside self-reported data. Finally, fostering collaboration with other rural hospitals and regional partners can create a supportive network for sharing best practices, resources, and challenges, thereby facilitating ongoing improvement.

## Conclusions

This simulation-based preparedness program illustrates how rural hospitals can rapidly enhance their capacity to manage mass casualty events through a structured, phased approach. The program successfully addressed key challenges in trauma care, communication, coordination, and system-level preparedness. Despite being implemented under extreme time constraints during an ongoing conflict, the intervention led to meaningful improvements as evidenced by external evaluations. Our experience highlights the value of simulation in uncovering latent safety threats and strengthening multidisciplinary collaboration. For similar settings, we recommend a comprehensive needs assessment, regular interdisciplinary simulations, and the integration of structured evaluation tools to guide improvement. This approach can serve as a practical model for other rural or resource-limited hospitals facing comparable preparedness challenges.
